# Maternal Sweeteners Intake Modulates Gut Microbiota and Exacerbates Learning and Memory Processes in Adult Male Offspring

**DOI:** 10.3389/fped.2021.746437

**Published:** 2022-01-07

**Authors:** Ana Laura de la Garza, Bianca Romero-Delgado, Alejandra Mayela Martínez-Tamez, Marcela Cárdenas-Tueme, Bianka Dianey Camacho-Zamora, Daniel Matta-Yee-Chig, Mónica Sánchez-Tapia, Nimbe Torres, Alberto Camacho-Morales

**Affiliations:** ^1^Universidad Autónoma de Nuevo León, Facultad de Salud Pública y Nutrición, Centro de Investigación en Nutrición y Salud Pública, Monterrey, Mexico; ^2^Universidad Autónoma de Nuevo León, Unidad de Nutrición, Centro de Investigación y Desarrollo en Ciencias de la Salud, Monterrey, Mexico; ^3^Universidad Autónoma de Nuevo León, Facultad de Ciencias Biológicas, Departamento de Biología Celular y Genética, Monterrey, Mexico; ^4^Universidad Autónoma de Nuevo León, Unidad de Genómica, Centro de Investigación y Desarrollo en Ciencias de la Salud, Monterrey, Mexico; ^5^Departamento de Fisiología de la Nutrición, Instituto Nacional de Ciencias Médicas y Nutrición Salvador Zubirán, Ciudad de México, Mexico; ^6^Universidad Autónoma de Nuevo León, Facultad de Medicina, Departamento de Bioquímica, Monterrey, Mexico; ^7^Universidad Autónoma de Nuevo León, Unidad de Neurometabolismo, Centro de Investigación y Desarrollo en Ciencias de la Salud, Monterrey, Mexico

**Keywords:** honey, sucrose, steviol glycosides, memory, gut microbiota

## Abstract

**Background:** There is increasing evidence that gut microbiota in offspring is derived in part from maternal environment such as diet. Thus, sweeteners intake including caloric or non-caloric during perinatal period can induce gut dysbiosis and program the offspring to develop cognitive problems later in life.

**Objective:** To determine the effect of maternal high-sweeteners intake during gestation and lactation on gut microbiota shifts in adult male offspring rats and the impact on cognitive dysfunction.

**Methods:** Thirty-four male pups from dams fed standard diet (Control-C, *n* = 10), high-sucrose diet (HS-C, *n* = 11), high-honey diet (Ho-C, *n* = 8), and high-stevia diet (HSt-C, *n* = 5) were fed standard diet after weaning, and body weight and food intake were recorded once a week for 26 weeks. Learning and memory tests were performed at week 23 of life using the Barnes maze. Fecal samples from the breastfeeding and adulthood periods were collected and analyzed by sequencing the 16S rRNA V3-V4 region of gut microbiota.

**Results:** Maternal high-sucrose and stevia diets programmed the male offspring, and changes in microbial diversity by Shannon index were observed after weaning (*p* < 0.01). Furthermore, maternal high-stevia diet programming lasted into adulthood. The increase of *Firmicutes* abundance and the decrease in phylum *Bacteroidetes* were significant in HS-C and HSt-C groups. This led to an increase in the Firmicutes/Bacteroidetes index, although only in HS-C group was statistically significant (*p* < 0.05). Of note, the downstream gram-negative *Bacteroidales* and the upregulation of the gram-positive *Clostridiales* abundance contribute to cognitive dysfunction.

**Conclusion:** These results suggest that dams fed a high-sucrose and stevia diets during gestation and lactation favor a deficient memory performance in adult male offspring rats through shifts gut microbiota diversity and relative abundance at several taxa.

## Introduction

In recent years, the gut–brain axis has been highlighted as a key pathway in the development of mental illnesses such as anxiety and depression (1), as well as cognitive disorders, including memory flexibility (2). In addition, recent studies indicate that bacteria colonization of the gastrointestinal tract through the microbial transmission of dams to offspring during early life modulates health in adult life (3). In fact, the deepest shifts on gut microbiota composition take place in childhood (4). During the first days of life, the newborn's intestine is colonized by Gram negative bacteria, which by consuming oxygen, generate an anaerobic environment and the relative abundance of *Lactobacillus* and *Bifidobacterium* genera (5). Thus, relatively minor changes occur, maintaining a stability of gut microbiota, although with the possibility of reshaping by the environment, including diet (6). This means that maternal nutritional exposures during pregnancy and breastfeeding could affect the microbial transmission to offspring modifying the microbiota ecosystem and setting health alterations associated with a dysbiotic microbiota (3).

Currently, the development of chronic diseases has been associated with the increase in sugar sweetened beverages (SSB) consumption (7). In a recent study of Hispanic families living in New York, the authors reported that 89% of parents and 66% of child aged 1–2 years old used SB regularly (8). In fact, more than 30% of pregnant women consume SSB, resulting in metabolic disorders not only in mothers but also in the childhood health (9). Thus, some authors report that the amount of sugar added in women from 25 to 34 years is between 7 and 11% of the total energy intake (7). Thereby, the non-caloric sweeteners consumption has been increasing due to low caloric content and its great acceptability as a substitute for sucrose (10). However, to date, there is controversy in the adverse health effects derived from both sucrose consumption and non-caloric sweeteners, including the steviol glycosides (11, 12). Precisely, murine models exposed to different non-nutritive sweeteners such as sucralose and aspartame develop glucose intolerance (13). In addition, diet with high glycemic index (GI) has an impact on learning and memory processes (14, 15). Nevertheless, other natural sweeteners such as bee honey have recently been highlighted despite having a caloric content; it is credited with beneficial health effects due to the phenolic compounds content (16, 17). For instance, a long-term honey consumption (52 wk) prevents memory loss compared to sucrose-fed rats (18). A tentative hypothesis proposes that the beneficial effects of bee honey consumption are related to shifts on gut microbiota. In fact, a prebiotic effect of honey allows changes in phyla *Actinobacteria* (genus: *Bifidobacterium*) and *Firmicutes* (genus: *Lactobacillus*) (19, 20). The most common phyla in the gut microbiota are *Firmicutes* and *Bacteroidetes*; however, other bacteria occur in lower quantities such as *Proteobacteria, Cyanobacteria, Verrucomicrobia*, and *Elusimicrobia* (21). Thus, considering the potential influence of maternal diet on cognitive performance in offspring (22, 23), we hypothesized that maternal high-sweeteners intake during gestation and lactation might promote gut microbiota dysbiosis leading to memory loss susceptibility in adult male offspring rats. Different experimental studies have shown the impact of maternal diet on bacterial diversity of the offspring, as well as specific changes in bacterial species (3, 24). Therefore, our objective was to evaluate the impact of maternal high-sweeteners intake during gestation and lactation on diversity and microbial composition of adult male offspring rats and its association with cognitive performance.

## Materials and Methods

### Diets

Dams were fed during the pregestational period with a cafeteria diet (372 kcal/100 g) which consisted of a mix of liquid chocolate, fried potatoes, bacon, biscuits, standard chow diet, and pork pate based on a 1:1:1:1:1:2 ratio. During gestation and lactation feeding period, all the diets were made with sweeteners mixed with standard chow diet formula (Rodent Lab Chow diet 5001; LabDiet, St. Louis, MO 63144). High-sucrose diet (HSD) consisted of a standard diet and condensed milk based on a 1:1.5 ratio (339 kcal/100 g). High-honey diet (HHD) consisted of a standard diet and bee honey based on a 1:1.15 ratio (339 kcal /100 g). High-stevia diet (HStD) consisted of a standard diet and organic steviol glycosides extract diluted in water based on a 1:1 ratio (335 kcal/100 g) (15). Honey from the flower of the avocado plant was used, which contains 38 g of fructose and 31 g of glucose per 100 g. The honey was obtained from the company Hermes Honey S.A. de C.V. located in Aguascalientes, Mexico. Condensed milk and organic steviol glycosides extract were obtained through a local supermarket. Otherwise, male offspring received the standard chow diet formula containing 335 kcal/100 g (Rodent Lab Chow diet 5008; LabDiet, St. Louis, MO 63144, USA).

### Animal Models

**Figure 1:**
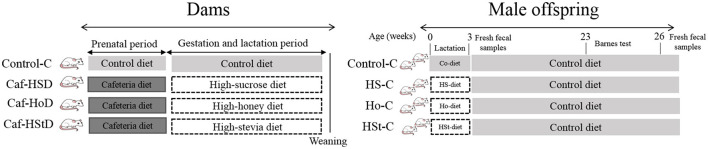
Experimental design.

Dams' experiments were performed with 12 female Wistar rats, 6 wk old, weighing 200–250 g from Tetrarium (Scientific, Technological, and commercial services S.A. de C.V. Monterrey, Mexico). The animals were acclimatized and fed a control diet for 2 wk. All animals were housed in polypropylene boxes in an environment of 21–22°C with 12-h light/dark cycles. Animals were randomly allocated in four groups: control-C group (*n* = 3), cafeteria-HSD group (*n* = 3), cafeteria-HHD group (*n* = 3), and cafeteria-HStD group (*n* = 3). The control-C group was fed a standard diet, and the cafeteria-HSD, cafeteria-HHD, and cafeteria-HStD groups were fed a cafeteria diet for 4 wk (pregestational period). Immediately after mating period, dams continued with the same diet (Control-C group) and the other rats were fed the high-sweetener isocaloric diets: high-sucrose diet (HSD), high-honey diet (HHD), and high-stevia diet (HStD) for 7 wk (gestation and lactation) (Figure 1). The body weight and food intake of the dams were recorded once a week.

Thirty-four male pups from dams fed standard diet (Control-C, *n* = 10), high-sucrose diet (HS-C, *n* = 11), high-honey diet (Ho-C, *n* = 8), and high-stevia diet (HSt-C, *n* = 5) were fed standard diet after weaning. Offspring rats had *ad libitum* water and food access, while body weight and food intake were recorded once a week. The Barnes maze test was performed at week 23 of life. Fresh fecal samples were collected by weeks 3 and 26 of life (Figure 1). At the end of the experimental period (week 26), pups were fasted overnight one night on an empty stomach and the next day fasting glucose was measured from a drop of blood collected from the tail vein. Finally, rats were sacrificed by decapitation and trunk blood was collected to obtain serum for further analyses. All the procedures were performed according to the national and institutional guidelines of the Animal Research Bioethics Committee of the Faculty of Public Health and Nutrition (CE 2/2019-13).

### Barnes Maze Test

The Barnes maze test (BMT) was used to assess spatial learning and memory performance, as previously described (15). The BMT was placed in a lighted and cold (16°C) room. Male rats were randomized to perform the tests in the maze at week 23 of life. The training lasted 5 days, and rats were trained with four trials per day. After efficient acquisition training, the short-term test was performed on the sixth day. Finally, a week later, the long-term test was conducted. Spatial learning in the BMT was assessed using escape latency (time to find the escape hole) and total errors (number of incorrect holes that were checked before the first encounter with the escape hole) on the platform. To analyze the number of errors, a semi-quantitative error scale was used as previously described (25).

### Fecal Sampling, DNA Extraction, and 16S Gene PCR Amplification

Fresh fecal samples were collected at the end of breastfeeding (week 3 of life) and during adulthood (week 26 of life) periods, early in the morning and after the overnight fasting period, by abdominal massage. Samples were collected in 15-ml Falcon tubes and immediately frozen at −80°C for further analyses. DNA from fecal samples was extracted using the QIAamp DNA Stool Mini Kit (Qiagen, Hilden, Germany), following supplier's instructions with a few modifications. DNA was measured and quantified by NanoDrop 8,000 (Thermo Scientific) and Picogreen fluorometer following the protocol. A linear regression was performed to calculate the final DNA concentration of each sample. All samples were quantified in triplicate. Values were expressed as ng/μl.

Variable regions 3–4 of the 16S rRNA gene were amplified using specific forward (5′ TCGTCGGCAGCGTCAGATGTGTATAAGAGACAGCCTACGGGNGGCWGCAG 3′) and reverse primers (5′ GTCTCGTGGGCTCGGAGATGTGTATAAGAGACAGGACTA CHVGGGTATCTAATCC 3′) containing the Illumina adapter overhang nucleotide sequences. PCRs were carried out using the following parameters: 3 min 95°C predenaturation; followed by 25 amplification cycles consisting of denaturation (30 s at 95°C), alignment (30 s at 63°C), and elongation (30 s at 72°C). The final elongation consisted of 5 min at 72°C. DNA concentration of amplicons of interest was determined by gel electrophoresis. DNA of each sample was pooled and purified with AMPure XP to remove primer dimers and other small mispriming products according to the manufacturer's specifications. An index PCR was then carried out to attach dual indices using a Nextera XT v2 kit.

### Illumina Mi-Seq Sequencing

Sequencing was performed on the Illumina MiSeq platform according to the manufacturer's instructions (Illumina, 16S Metagenomic Sequencing Library Preparation). Libraries were demultiplexed using Illumina's bcl2fastq 1.8.4 software (Illumina), and reads were processed with custom Python scripts to sort them into samples, removing barcode and amplicon primers sequence. For taxonomic composition analysis, custom Python scripts in the Quantitative Insights into Microbial Ecology (QIIME, San Diego, CA, USA) software pipeline 1.9 were used to process the sequencing files. The sequence outputs were filtered for low-quality sequences (defined as any sequences that are <200 bps or >600 bps, sequences with any nucleotide mismatches to either the barcode or the primer, sequences with homopolymer runs >6, sequences with an average quality score of <30, and sequences with ambiguous bases >0) and were truncated at the reverse primer. Sequences were chimera checked with Chimera Slayer, and chimeric sequences were filtered out. Analysis started by clustering sequences within a percent sequence similarity into operational taxonomic units (OTUs) with a 97% similarity threshold. Thus, 100, 100, 100, 90.77, and 73.42% of the reads were assigned to the phylum, class, order, family, and genus level, respectively. Alpha diversity measurements (Shannon) were calculated. Weighted and unweighted UniFrac distances were used to perform the principal coordinate analysis (PCoA) for beta diversity.

### Statistical Analysis

Statistical analyses were conducted using SPSS 20.0 (SPSS Inc., Chicago, IL, USA). Results are expressed as mean ± SEM. Statistically significant differences between experimental groups (Control-C, HS-C, Ho-C, and HSt-C) were determined by one-way analysis of variance (ANOVA) test followed by Dunnett's *post-hoc* test and Kruskall Wallis non-parametric equivalent test. *T*-test for related samples was performed to compare microbial community composition between breastfeeding and adulthood. The correlation between *Firmicutes*/*Bacteroidetes* index with glucose levels and spatial learning was performed using the Spearman correlation coefficient. A level of probability of *p* < 0.05 was set as statistically significant. Sample sizes can be found in the figure legends, where n represents the number of animals used for each analysis.

## Results

### Maternal High-Sweeteners Intake During Gestation and Lactation Influences the Birth Weight and Body Weight Gain of Male Pups

**Figure 2:**
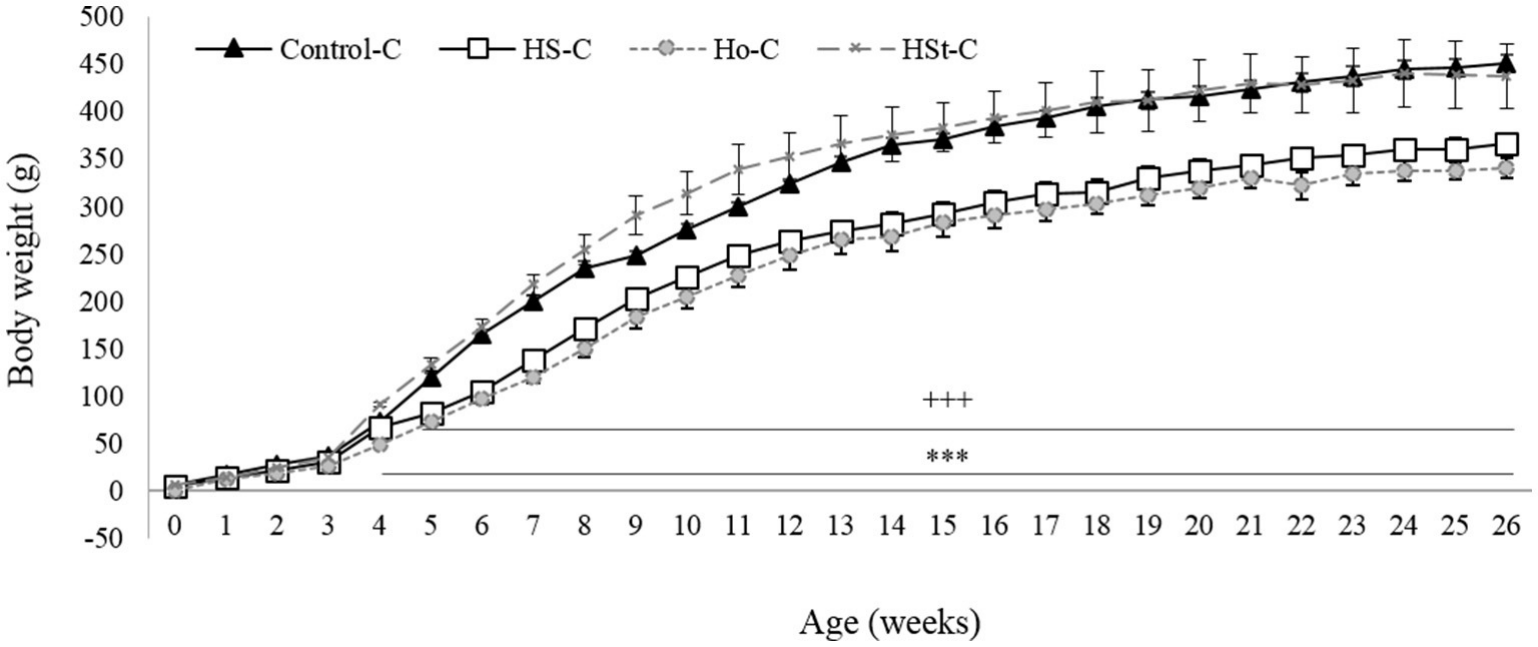
Effect of maternal high-sweeteners diet on body weight in 26-week-old male offspring. All the results are expressed as the mean ± SEM. Statistical analyses were performed using one-way ANOVA and Dunnett's test *post-hoc*. Control-C (*n* = 10), HS-C (*n* = 11), Ho-C (*n* = 8), HSt-C (*n* = 5). ^+++^ *p* < 0.001 (Control-C vs. HS-C); ^***^ *p* < 0.001 (Control-C vs. Ho-C).

**Figure 3:**
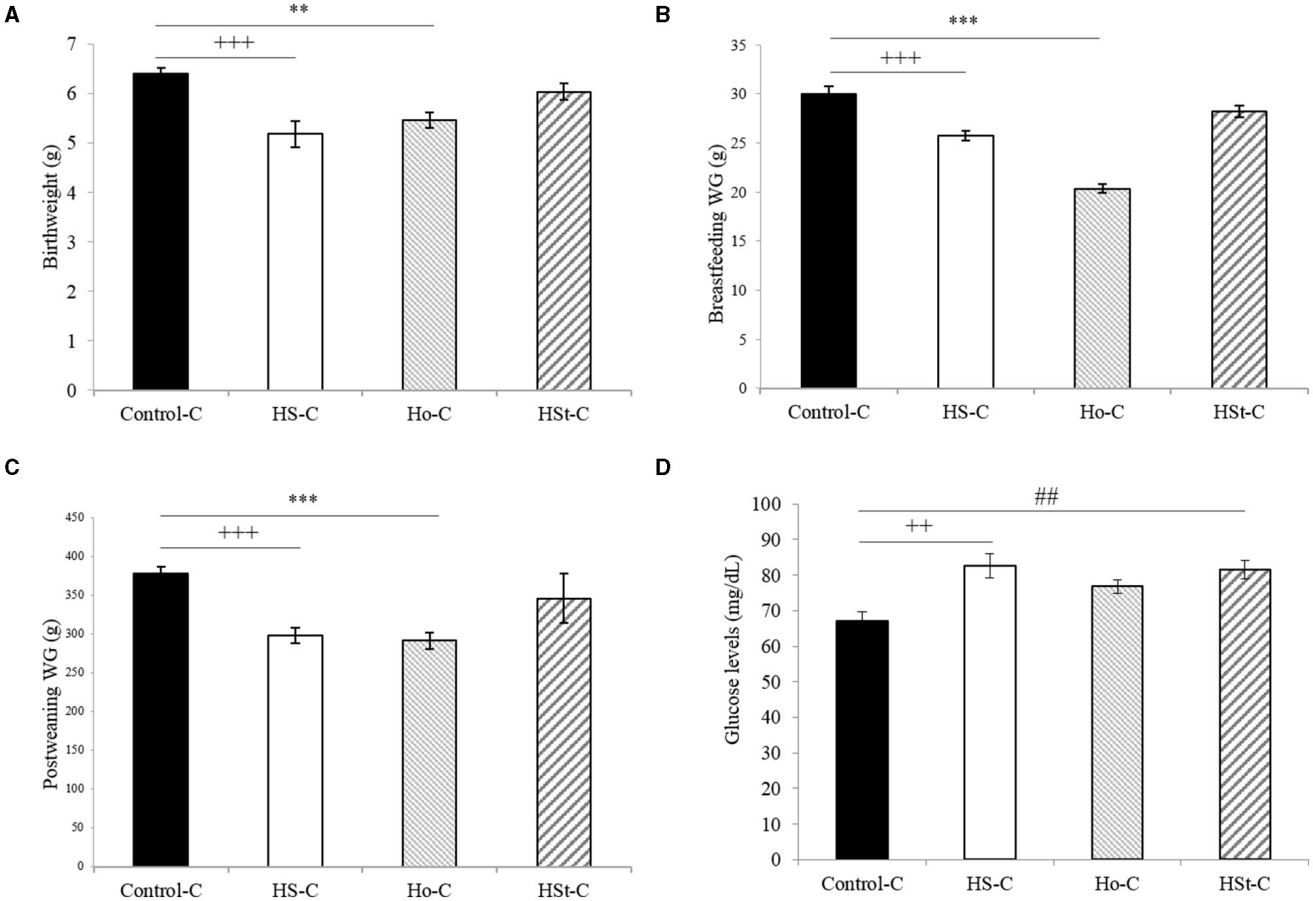
Effect of maternal high-sweeteners diet on birthweight (g) **(A)**, breastfeeding weight gain (g) **(B)**, postweaning weight gain (g) **(C)**, and blood glucose levels (mg/dl) **(D)** in male offspring rats. All the results are expressed as the mean ± SEM. Statistical analyses were performed using one-way ANOVA and Dunnett's test *post-hoc*. Control-C (*n* = 10), HS-C (*n* = 11), Ho-C (*n* = 8), HSt-C (*n* = 5). ^++^ *p* < 0.01, ^+++^ *p* < 0.001 (Control-C vs HS-C); ^**^ *p* < 0.01, ^***^ *p* < 0.001 (Control-C vs Ho-C); ## *p* < 0.01 (Control-C vs. HSt-C).

Figure 2 shows the weight gain of male pups throughout the experimental period. Statistically significant decrease in birth weight were found between HS-C (5.18 ± 0.27 g; *p* < 0.001) and Ho-C (5.46 ± 0.15; *p* < 0.01) groups compared with the Control-C group (6.41 ± 0.11 g) (Figure 3A). Likewise, body weight gain during the 3 weeks of the breastfeeding period was significantly lower in the HS-C (25.77 ± 0.52 g) and Ho-C (20.37 ± 0.42 g) groups compared with the Control-C group (*p* < 0.001) (Figure 3B).

In addition, after the breastfeeding period, male offspring of HS-C and Ho-C groups continued to gain less weight compared with the Control-C group, showing significant results from week 4 (Ho-C group) and 5 (HS-C group) to week 26 of life (Figure 2). Therefore, postweaning weight gain (3^rd^ to 26^th^ week) was lower in the HS-C group (298.38 ± 10.34 g) and Ho-C group (291.34 ± 10.20 g) compared with the Control-C group (*p* < 0.001), whereas no significant differences were found when Control-C (377.72 ± 8.71 g) and HSt-C (346.21 ± 32.03) groups were compared (*p* = 0.38) (Figure 3C).

Otherwise, we found a significant increase in the fasting glucose levels of male offspring rats in the HS-C (82.60 ± 3.40 mg/dl) and HSt-C (81.60 ± 2.63 mg/dl) groups when compared with the Control-C (67.20 ± 2.47 mg/dl; *p* < 0.01) (Figure 3D).

### The Effect of Maternal High-Sweeteners Intake During Gestation and Lactation on Learning and Memory Performance in Adult Male Offspring Rats

**Figure 4:**
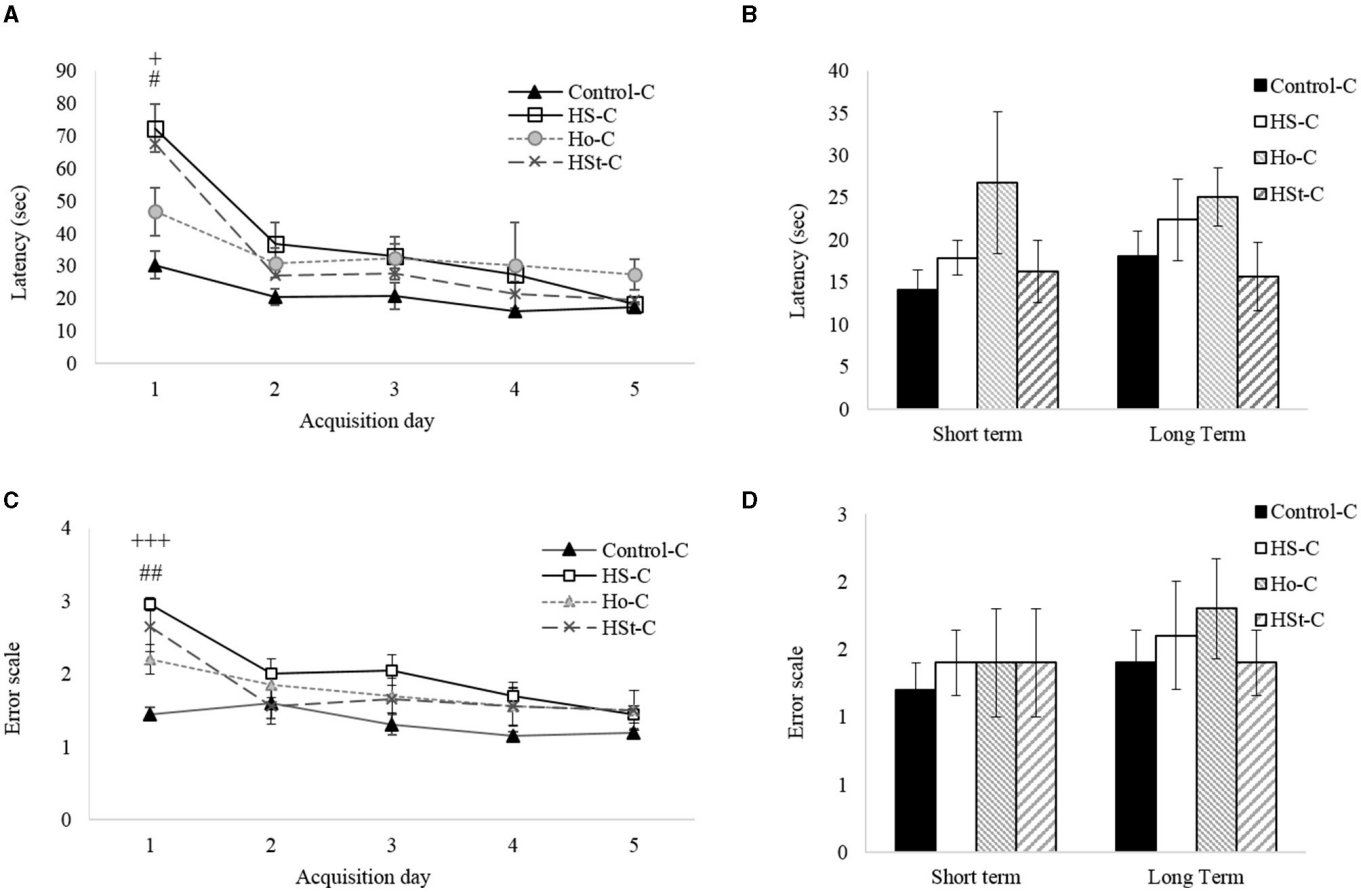
Barnes maze test. Effect of maternal high-sweeteners diet on latency (s) **(A, B)**, and error scale **(C, D)** in male offspring rats. All the results are expressed as the mean ± SEM. Statistical analyses were performed using one-way ANOVA and Dunnett's test post-hoc. Control-C (*n* = 5), HS-C (*n* = 5), Ho-C (*n* = 5), HSt-C (*n* = 5). ^+^ *p* < 0.05, ^+++^ *p* < 0.001 (Control-C vs HS-C); # *p* < 0.05, ## *p* < 0.01 (Control-C vs HSt-C).

For the analysis of learning and spatial memory, training and short- and long-term tests were carried out in the Barnes Maze platform (Figure 4). We found that the Control-C group by day 1 of training spent an average of 30.28 ± 4.25 s to find the escape hole on the platform. On the other hand, when evaluating the average time to find the escape hole per groups, it was observed that the HS-C group (72.28 ± 7.35 s) displays defective performance to find the exit from the platform when compared with the Control-C group (*p* < 0.05). Similar results were found between the Control-C group and HSt-C group (67.65 ± 8.52 s). Nevertheless, although the average time of the Ho-C group (46.72 ± 7.37 s) was longer than the Control-C group, no significant differences were observed between groups. Following the training schedule, no significative differences were observed between the HS-C, HSt-C, and Ho-C groups and Control-C group (Figure 4A).

Likewise, when analyzing the error scale during training days, the Control-C group preserves the error scale throughout the training schedule (Figure 4C). Of note, as we found in the latency analysis, a significant difference in the scale of error at the first day of training (*p* < 0.05) was found between the HS-C (2.95 ± 0.09) and HSt-C (2.65 ± 0.35) groups compared with the Control-C group (1.45 ± 0.09).

Once the training was completed, the male pups of the HS-C, HSt-C, and Ho-C and Control-C groups performed the short-term (1 day after training) and long-term (1 week after the short-term test) tests. We found no significant differences between groups (Figures 4B,D).

### Maternal High-Sweeteners Intake During Gestation and Lactation Modifies Gut Microbial Profile

Gut microbiota composition varied between the HS-C, Ho-C, and HSt-C groups when compared with Control-C group. Therefore, we generated relative abundance (%) values for each animal and present the mean ± SEM per group. In addition, although samples were evaluated from breastfeeding and adulthood periods, changes in phylum, order, family, and genus levels were analyzed only in adult male rats.

**Figure 5:**
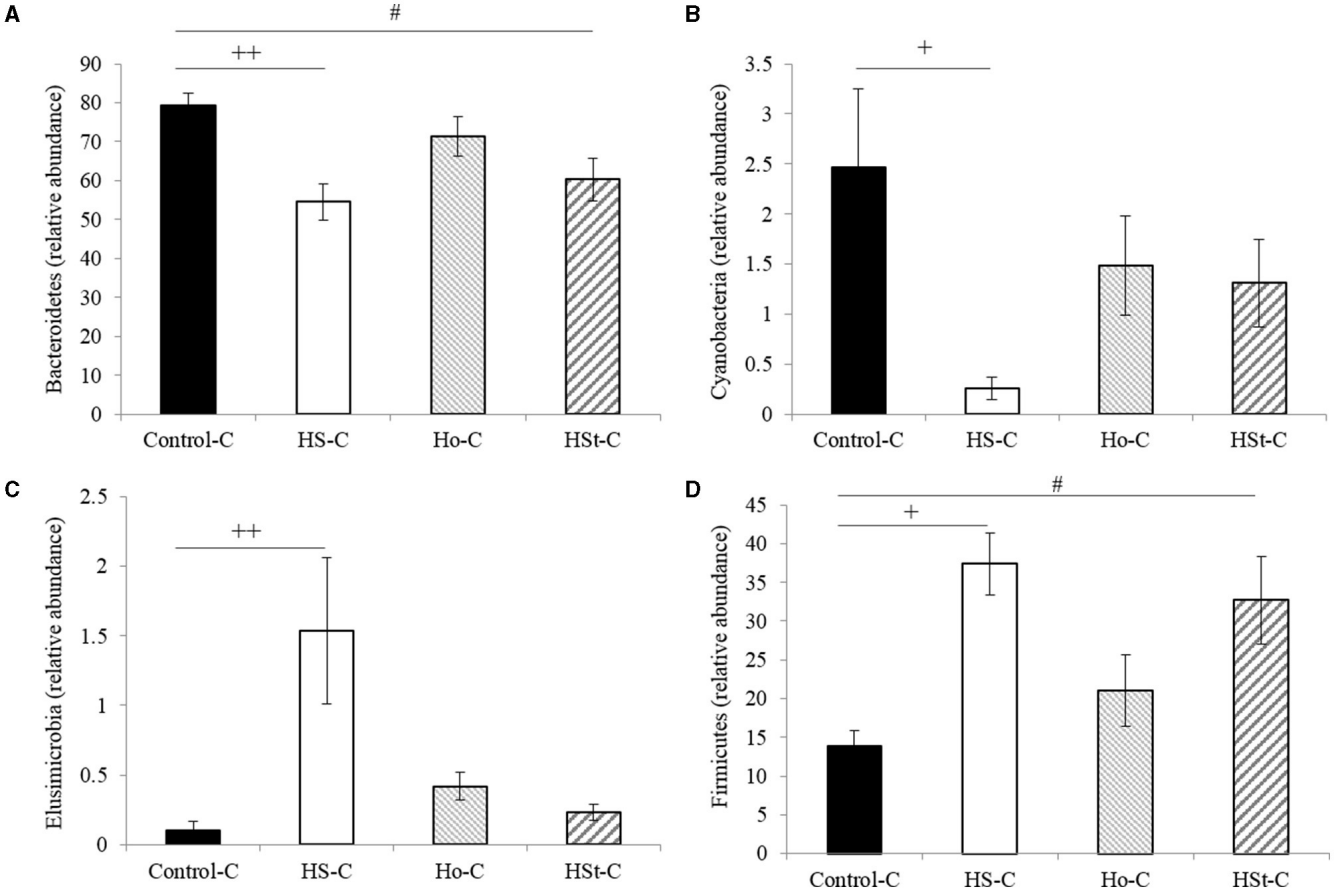
Relative abundance of bacterial phyla of adult male rats. All the results are expressed as relative abundance (%) of sequences and presented as mean ± SEM. Statistical analyses were performed using one-way ANOVA and Dunnett's test post-hoc or Kruskall Wallis non-parametric equivalent test. Control-C (*n* = 5), HS-C (*n* = 5), Ho-C (*n* = 4), HSt-C (*n* = 5). ^+^ *p* < 0.05, ^++^ *p* < 0.01 (Control-C vs HS-C); ^#^ *p* < 0.05 (Control-C vs HSt-C).

At the phylum level, maternal high-sweeteners diets decreased *Bacteroidetes* and *Cyanobacteria*, while *Elusimicrobia* and *Firmicutes* increased (Figure 5). Thus, maternal high-sucrose diet during gestation and lactation significantly decreased the relative abundance of *Bacteroidetes* (*p* < 0.001) and *Cyanobacteria* (*p* < 0.05) (Figures 5A,B). Conversely, HS-C group significantly increased the relative abundance of *Elusimicrobia* (*p* < 0.01) and *Firmicutes* (*p* < 0.05) (Figures 5C,D). According to previous results, in male offspring rats from dams fed high-stevia diet, *Bacteroidetes* and *Cyanobacteria* decreased, while *Elusimicrobia* and *Firmicutes* increased compared with the Control-C group. However, only significant results were obtained in *Bacteroidetes* and *Firmicutes* phylum (*p* < 0.05). Interestingly, Ho-C group did not present significant differences compared with the Control-C group at the phylum level (Figure 5).

**Figure 6:**
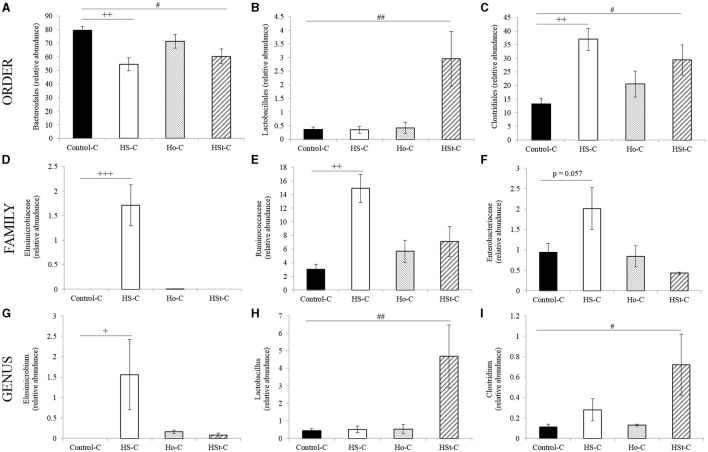
Relative abundance of gut bacterial taxa at the different level **(A–C)** at the order level; **(D–F)** at the family level; **(G–I)** at the genus level of adult male rats. All the results are expressed as relative abundance (%) of sequences and presented as mean ± SEM. Statistical analyses were performed using one-way ANOVA and Dunnett's test post-hoc or Kruskall Wallis non-parametric equivalent test. Control-C (*n* = 5), HS-C (*n* = 5), Ho-C (*n* = 4), HSt-C (*n* = 5). ^+^ *p* < 0.05, ^++^ *p* < 0.01 (Control-C vs. HS-C); ^#^ *p* < 0.05 (Control-C vs. HSt-C).

At the order level, maternal high-sucrose diet significantly decreased the relative abundance of *Bacteroidales* (Phylum: *Bacteroidetes*) (*p* < 0.01) and increased *Clostridiales* (Phylum: *Firmicutes*) (*p* < 0.01) when compared with Control-C group (Figures 6A,C). Also, an increase in the relative abundance of *Lactobacillales* (Phylum: *Firmicutes*) was found in the HSt-C group compared with the Control-C group (*p* < 0.01) (Figure 6B). At the family level, a significant increase in the *Elusimicrobiaceae* (Phylum: *Elusimicrobia*) (*p* < 0.001), *Ruminococcaceae* (Phylum: *Firmicutes*; Order: *Clostridiales*) (*p* < 0.01), and *Enterobacteriaceae* (Phylum: *Proteobacteria*) (*p* = 0.057) was found only in the HS-C group compared with the Control-C group (Figures 6D–F).

Finally, at the genus level, the abundance of *Elusimicrobium* (Phylum: *Elusimicrobia*; Family: *Elusimicrobiaceae*) was significantly increased in the HS-C group compared with the Control-C group (*p* < 0.05) (Figure 6G). In contrast, the relative abundance of *Lactobacillus* (Phylum: *Firmicutes*; Order: *Lactobacillales*) and *Clostridium* (Phylum: *Firmicutes*; Order: *Clostridiales*) was significantly higher in the HSt-C group compared with the Control-C group (*p* < 0.01 and *p* < 0.05, respectively) (Figures 6H,I).

### Impact of Maternal High-Sweeteners Diet on the Bacterial Diversity of Male Pups During Breastfeeding and the Reshaping After 23 Weeks Fed Control Diet

**Figure 7:**
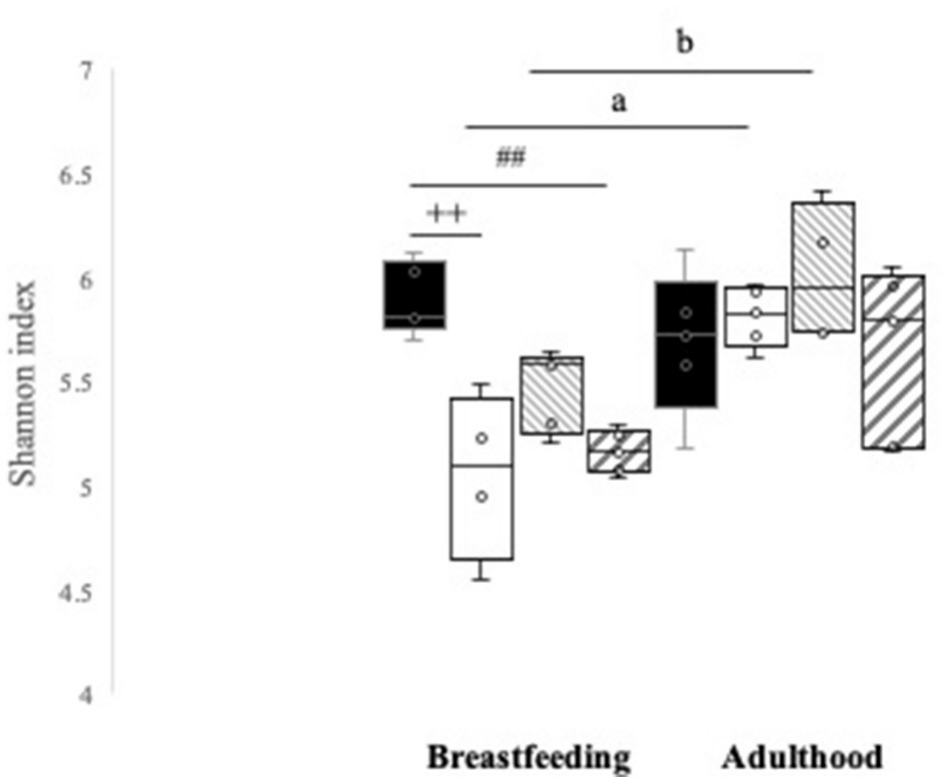
Maternal high-sweeteners diet modifies the gut microbiota diversity by Shannon index. All the results are expressed as relative abundance (%) of sequences and presented as mean ± SEM. Control-C, HS-C, Ho-C, HSt-C. Statistical analyses were performed using one-way ANOVA and Dunnett's test *post-hoc* or Kruskall Wallis non-parametric equivalent test. Differences of means per group between the two periods were analyzed by *T*-test for related samples. Control-C (*n* = 5), HS-C (*n* = 5), Ho-C (*n* = 4), HSt-C (*n* = 5). ^++^ *p* < 0.01 (Control-C vs. HS-C); ^*##*^ *p* < 0.01 (Control-C vs. HSt-C); ^*a*^ *p* < 0.05 (HS-C); ^*b*^ *p* < 0.05 (Ho-C).

In Figure 7, the Shannon index by groups is observed when analyzing fecal samples during breastfeeding and in adulthood of male pup rats. Results show that during breastfeeding, the Control-C group (5.89 ± 0.07) exhibits greater α-diversity compared with the HS-C (5.05 ± 0.20) and HSt-C (5.16 ± 0.46) groups (*p* < 0.01). However, unexpectedly, no difference was observed in the Ho-C group compared with Control-C group (5.46 ± 0.08).

Nevertheless, no significant changes in the α-diversity of adulthood were identified between the HS-C, HSt-C, and Ho-C groups when compared with Control-C group. However, significant changes were observed when comparing the Shannon index between breastfeeding and adulthood periods per groups. This indicates that both the HS-C group (*p* = 0.017) and the Ho-C group (*p* = 0.036) reshape the gut microbiota after 23 wk fed control diet. However, it is noteworthy that in the HSt-C group, no significant differences were observed during breastfeeding and adulthood in the Shannon index (*p* = 0.11) (Figure 7).

**Figure 8:**
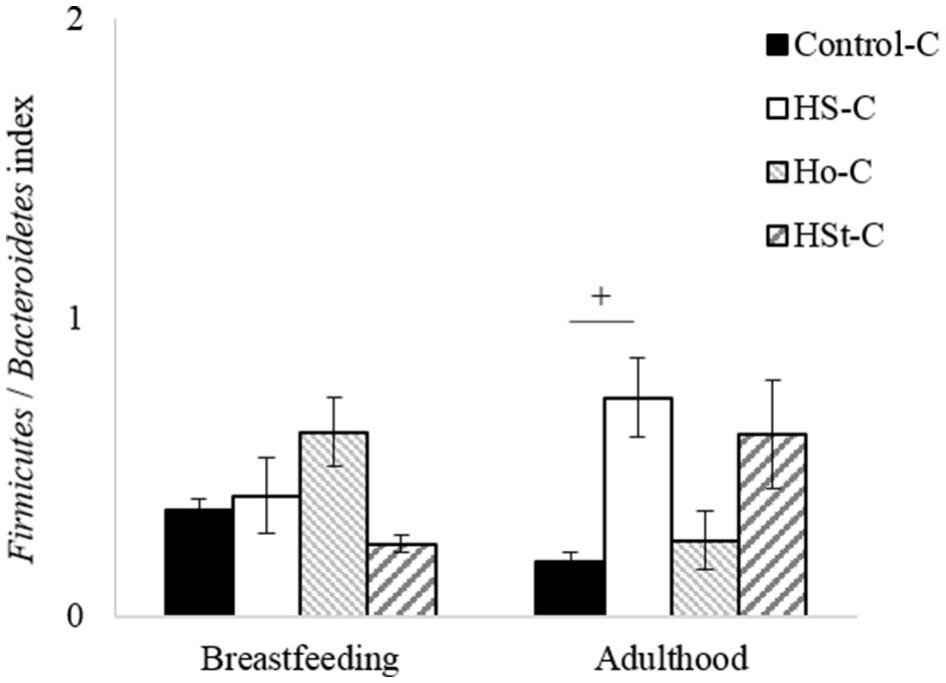
*Firmicutes*/*Bacteroidetes* index of male pups at breastfeeding and adulthood periods. All the results are expressed as relative abundance (%) of sequences and presented as mean ± SEM. Statistical analyses were performed using one-way ANOVA and Dunnett's test *post-hoc* or Kruskall Wallis non-parametric equivalent test. Control-C (*n* = 5), HS-C (*n* = 5), Ho-C (*n* = 4), HSt-C (*n* = 5). ^+^ *p* < 0.05 (Control-C vs. HS-C).

Moreover, the Firmicutes/Bacteroidetes index was calculated (Figure 8). Thus, no significant differences were found between groups in the breastfeeding period. However, a significant difference was observed between Control-C group (0.18 ± 0.03) and HS-C group (0.73 ± 0.13) in adulthood (*p* < 0.05). This change indicates a considerable 4.05-fold increase in the HS-C group.

### Modulatory Effect of Maternal High-Sweeteners Diet on Firmicutes/Bacteroidetes Index Contributes to the Cognitive Dysfunction in Adult Male Rats

**Figure 9:**
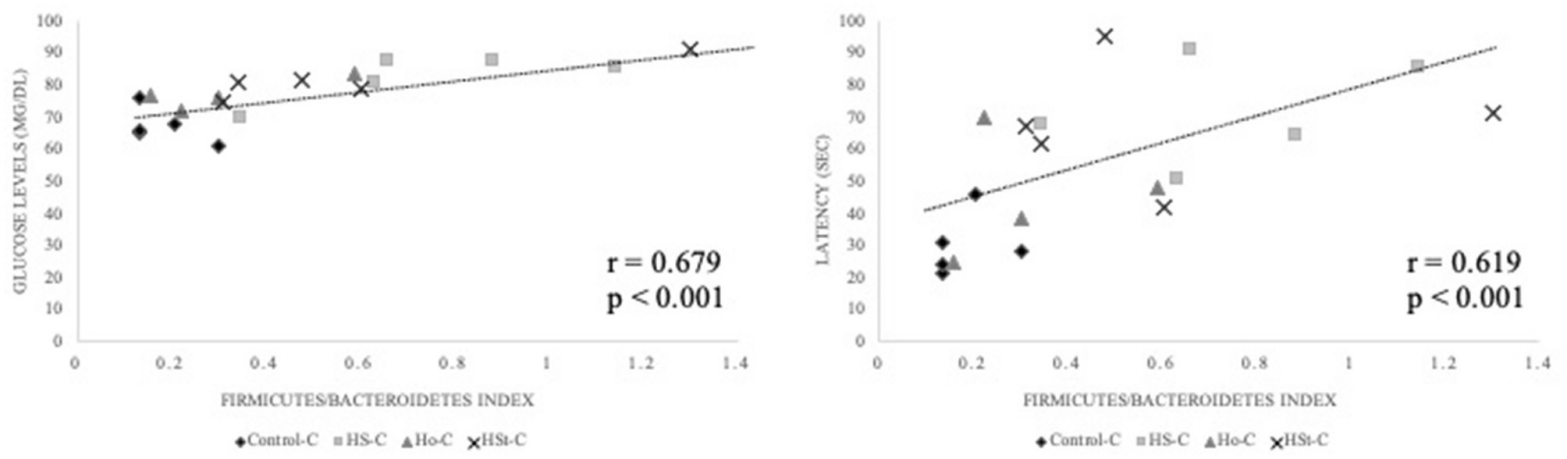
Correlations between the F/B index with glucose levels (mg/dl) and latency (s) in Barnes maze of adult male offspring rats. Spearman's rank correlations were conducted taking into consideration Control-C (*n* = 5), HS-C (*n* = 4), Ho-C (*n* = 4), and HSt-C (*n* = 5). Results were considered statistically significant when *p* < 0.05.

Significant and positive correlations were found between *Firmicutes/Bacteroidetes* (F/B) index calculated in adult male rats fed control diet, and glucose levels (mg/dl) (*r* = 0.679; *p* < 0.001) and latency (s) in Barnes maze (*r* = 0.619; *p* < 0.001) (Figure 9).

## Discussion

Epidemiological and experimental studies have shown a relationship between maternal environment during the perinatal period and the risk of developing chronic diseases in adult offspring (26). Reports using animal models documented the impact of the maternal diet on the susceptibility to developing metabolic disorders, such as obesity, in adult offspring (27–30). Therefore, in a recent review, Ribaroff et al. analyze the effects of the maternal high-fat diet on offspring health, confirming effects on adiposity and final body weight, whereas no changes were found in the pups' birthweight (31). Otherwise, maternal high-sucrose diet has been related to higher birthweight in male offspring (32). However, in addition to the effects on metabolic disorders in the offspring, maternal exposure to high caloric diets favors behavioral changes such as depression (33), anxiety (18), as well as learning and memory (34). Furthermore, some studies report that high-sucrose or high-fructose corn syrup diets can impact learning and memory processes, regardless of the obesity development (35). These changes might potentially be associated with the effect of the maternal diet on offspring gut microbiome (24). Based on this proposal, our study analyzed the shifts on gut microbiota of male offspring rats associated to maternal high-sweeteners diets during gestation and lactation and the impact on different metabolic parameters and cognitive dysfunction.

In recent years, the role of gut microbiota as responsible for the relationship between high-sucrose diet and glucose intolerance has been highlighted (36). In contrast, a study performed with dyslipidemic rats reported that supplementation with honey from Mimosa quadrivalvis L. increased glucose tolerance (19). Honey is a caloric sweetener (3.4 kcal/g) composed mainly of glucose and fructose, and it is also a source of flavonoids and phenolic acids, and its composition depends on the type of flower used by bees (17). Conversely, in recent years, the effect of non-caloric sweeteners on the regulation of glucose levels has also been evaluated. The results to date are controversial, finding that some artificial sweeteners induce glucose intolerance (37). This may be due to the type of non-caloric sweetener consumed, its absorption and transport in the small and large intestines, as well as shifts in gut microbiota (38). For example, in a crossover trial with healthy subjects, after the consumption of 1 g of stevia diluted in 300 ml of water, no significant changes were observed in postprandial glucose levels (12).

Thus, in our study, we found high glucose levels in HS-C and HSt-C groups compared with the Control-C group; in addition, a correlation between glucose levels and the F/B index was found. The F/B index has been widely used as a marker of obesity; however, there are contradictory data when associating the F/B index with a health status (39). Different studies have defined dysregulations in Firmicutes and Bacteroidetes phyla associated with metabolic changes such as obesity (21). However, these changes have also been associated with cognitive impairments (40). Surprisingly, in our study, the changes found in *Firmicutes* and *Bacteroidetes* phyla were in adulthood regardless of body weight gain.

Likewise, in addition to the correlation between F/B index with glucose levels, a positive association was also found between F/B index with the escape latency, an indicator of spatial memory. On this context, the offspring of the HS-C and HSt-C groups showed the longest time to find the escape hole during the Barnes maze test. Several studies have reported that high-sucrose diet is related not only to glucose intolerance but also to cognitive defects (14, 18, 41). Our study provides evidence that maternal high-sucrose diet promotes a shift on gut microbiota of adult rats, which correlates to changes in glucose levels and memory loss. Likewise, in recent years, the use of non-caloric sweeteners such as steviol glycosides has been preferred to “decrease” caloric intake (12). However, in our study, maternal high-stevia diet programmed the offspring by altering glucose levels and inducing memory loss. Thus, although the male pups from dams fed high sucrose and steviol glycosides diets did not show increase in body weight, we propose that HS-C and HSt-C groups presented shifts on gut microbiota that may be associated with high glucose levels and cognitive deficits.

Experimental evidence have documented that selective dietary ingredients modulate the gut microbiota, as well as its relationship with neurocognitive dysfunction (40). For instance, at the order level, high-sucrose diet increases *Clostridiales* (Phylum: *Firmicutes*) and decreases *Bacteroidales* (Phylum: *Bacteroidetes*), which were correlated with a cognitive deficit (1). Similar results were found in our study in HS-C group compared with the Control-C group. These results show an increase in bacterial communities that have been associated with learning and memory defects. In addition, in our study, we also found an increased abundance of *Clostridiales* and a decrease in *Bacteroidales* in adult male rats from dams fed high-stevia diet. Unlike the high-sucrose diet, to date, the relationship between *Stevia rebaudiana* consumption and changes in gut microbiota is not entirely clear. A report identified that administered water with 2.5% steviol glycosides to male wistar rats leads to lower α-diversity, in contrast to other sweeteners such as sucrose or honey (42). Furthermore, it has been reported that *Bacteroides* are, at the genus level, the group of bacteria that hydrolyze stevioside and rebaudioside A to steviol. However, the use of steviol glycosides as a substrate for *Clostridium* and *Lactobacilli* was not found (38). In this regard, in our study, we found that only adult male offspring of dams fed high-stevia diet significantly increase the relative abundance of *Lactobacillus* and *Lactobacillales* at the genus and order levels, respectively.

In contrast, other studies have reported that the *Lactobacilli* genus uses steviol glycosides very poorly (43). Furthermore, in another study, the authors reported that in the presence of stevia sweeteners stevioside and rebaudioside A, the growth of the *Lactobacillus reuteri* species is inhibited (44). *Lactobacillus* have been reported to facilitate the transport of short chain fatty acids (45), which seem to be associated with cognitive performance (2). Nevertheless, although our study did not find an increase in *Lactobacillales* in the Ho-C group, other authors have reported an increase in *Lactobacillus* spp. in dyslipidaemic rats supplemented with honey (19). The difference between these reported data may be due to the fructans content in honey or Stevia rebaudiana. In fact, fructans enhance the growth of *Lactobacilli* and *Bifidobacteria*, key bacteria in gut health (38). However, although no difference of *Lactobacillus* (Order: *Lactobacillales*) was found in Ho-C group, maternal high-honey diet programmed the male offspring to show greater bacterial diversity than HS-C and HSt-C groups.

Experimental research have highlighted the shifts in bacterial diversity, caused by diet, and its relationship with health (3, 42). As an example, exposure to western or cafeteria diets affects bacterial diversity leading to diet-related diseases (46). Bacteria adapt to environment and can dynamically interact with each other and the host, contributing to the host's health (47). Therefore, when evaluating the reshaping fecal gut microbiota in male adult offspring fed control diet for 23 weeks, it was observed that the Ho-C group increased bacterial diversity significantly. Likewise, HS-C group also restored the bacterial diversity by significantly increasing the Shannon index in adulthood compared to breastfeeding period. However, in the HSt-C group, no significant changes were found in Shannon index between both periods. This suggests that in addition to the loss of microbial diversity due to the maternal diet, there is a progressive loss due to the programming of stevia diet early in life, related to the breastfeeding period. The foregoing may be related to the effects found in spatial memory of HSt-C group regardless of the increase of *Lactobacillales*.

Finally, although there is few evidence on the phylum *Elusimicrobia*, an association between the genus *Elusimicrobium* and the decrease in blood glucose levels in diabetic rats has been reported (48). In this regard, although there is no evidence of the relationship with cognitive defects, our results are similar in the increase in blood glucose levels in the HS-C group and the positive correlation with the relative abundance of *Elusimicrobiaceae* family. In addition, it was also found that the HS-C group increases relative abundance of *Enterobacteriaceae*, which has been reported to be associated with gut and brain inflammation (35). Although no links between *Elusimicrobiaceae* and *Enterobacteriaceae* and memory defects have been found, there is evidence that both hyperglycemia and inflammation may be related to brain-related diseases (34, 49). Therefore, one of the mechanisms involved in the development of cognitive diseases may be the bacteria presence such as *Enterobacteriaceae* and *Elusimicrobiaceae* that regulate inflammatory and glycemic process in the host.

Thus, in this study, the downstream *Bacteroidales* and the upregulation of *Clostridiales* abundance at the order level were a key pathway for the cognitive dysfunction. In addition, reshaping gut microbiota is possible in adulthood, but bacterial diversity increased only in HS-C and Ho-C groups, highlighting the long-term effect of maternal high-stevia diet on the gut microbiota of the offspring.

It is noteworthy that our study reports interesting data on the effect of maternal high-sweeteners diet on the bacterial abundance and diversity of adult male offspring rats. Furthermore, these significant changes in male pups' microbiota may be responsible for the effects on learning and memory processes. This suggests that shifts on gut microbiota through the maternal diet exposure may be the mechanism involved in the development of cognitive problems in adult offspring. On the other hand, although tests were also carried out on female pups and the results have already been published previously (15), the microbiota analysis was only performed on male pups. However, both female and male offspring were found to have effects on long-term learning and memory.

In summary, the pups of dams fed high-sucrose and stevia diets induced hyperglycemia and experience defective memory performance in adult male offspring rats, regardless of weight gain. One of the mechanisms involved in these effects may be changes in the microbial diversity of male offspring caused by the maternal diet.

## Data Availability Statement

The data presented in the study are deposited in the Dryad repository with accession number 10.5061/dryad.7sqv9s4th.

## Ethics Statement

The animal study was reviewed and approved by Animal Research Bioethics Committee of the Faculty of Public Health and Nutrition (CE 2/2019-13).

## Author Contributions

AdlG: conceptualization. BR-D, AM-T, BC-Z, and DM-Y-C: investigation. MC-T and MS-T: methodology. AdlG, NT, and AC-M: supervision. AdlG and AC-M: visualization. AdlG, BR-D, AM-T, MC-T, BC-Z, DM-Y-C, MS-T, NT, and AC-M: writing—review and editing. All authors contributed to the article and approved the submitted version.

## Funding

This research was funded by Programa de apoyo a la investigación científica y tecnológica (PAICYT), UANL, Grant Number SA754-19. Also, we acknowledge Consejo Nacional de Ciencia y Tecnología (CONACYT) México, for the grant awarded to BR-D.

## Conflict of Interest

The authors declare that the research was conducted in the absence of any commercial or financial relationships that could be construed as a potential conflict of interest.

## Publisher's Note

All claims expressed in this article are solely those of the authors and do not necessarily represent those of their affiliated organizations, or those of the publisher, the editors and the reviewers. Any product that may be evaluated in this article, or claim that may be made by its manufacturer, is not guaranteed or endorsed by the publisher.
